# Postembryonic Establishment of Megabase-Scale Gene Silencing in Nucleolar Dominance

**DOI:** 10.1371/journal.pone.0001157

**Published:** 2007-11-07

**Authors:** Olga Pontes, Richard J. Lawrence, Manuela Silva, Sasha Preuss, Pedro Costa-Nunes, Keith Earley, Nuno Neves, Wanda Viegas, Craig S. Pikaard

**Affiliations:** 1 Department of Biology, Washington University, St. Louis, Missouri, United States of America; 2 Centro de Botânica Aplicada à Agricultura, Instituto Superior de Agronomia, Technical University of Lisbon, Lisboa, Portugal; 3 Secção Autónoma de Biotecnologia, Faculdade de Ciências e Tecnologia, Universidade Nova de Lisboa, Caparica, Portugal; Institut Curie, France

## Abstract

Nucleolar dominance is an epigenetic phenomenon in plant and animal genetic hybrids that describes the expression of 45S ribosomal RNA genes (rRNA genes) inherited from only one progenitor due to the silencing of the other progenitor's rRNA genes. rRNA genes are tandemly arrayed at nucleolus organizer regions (NORs) that span millions of basepairs, thus gene silencing in nucleolar dominance occurs on a scale second only to X-chromosome inactivation in female mammals. In *Arabidopsis suecica*, the allotetraploid hybrid of *A. thaliana* and *A. arenosa*, the *A. thaliana* –derived rRNA genes are subjected to nucleolar dominance and are silenced via repressive chromatin modifications. However, the developmental stage at which nucleolar dominance is established in *A. suecica* is currently unknown. We show that nucleolar dominance is not apparent in seedling cotyledons formed during embryogenesis but becomes progressively established during early postembryonic development in tissues derived from both the shoot and root apical meristems. The progressive silencing of *A. thaliana* rRNA genes correlates with the transition of *A. thaliana* NORs from a decondensed euchromatic state associated with histone H3 that is trimethylated on lysine 4 (H3K4me3) to a highly condensed heterochromatic state in which the NORs are associated with H3K9me2 and 5-methylcytosine-enriched chromocenters. In RNAi-lines in which the histone deacetylases HDA6 and HDT1 are knocked down, the developmentally regulated condensation and inactivation of *A. thaliana* NORs is disrupted. Collectively, these data demonstrate that HDA6 and HDT1 function in the postembryonic establishment of nucleolar dominance, a process which recurs in each generation.

## Introduction

Epigenetic phenomena reflect alternative gene expression states that are maintained through multiple rounds of mitosis, and sometimes through meiosis. The alternative expression states are not due to mutation or changes in gene sequence and are reversible to varying degrees [1], [2]. One of the first epigenetic phenomena described was nucleolar dominance, initially known as differential amphiplasty [3]. The phenomenon occurs in genetic hybrids and has cytologically visible consequences on chromosome morphology [3]. First discovered in plants, nucleolar dominance occurs in organisms as diverse as fruit flies, frogs, mammals and marine copepods [3], [4], [5], [6], [7]. Nucleolar dominance is typically studied in interspecific hybrids (hybrids formed between species within the same genus) and occurs independent of maternal or paternal effects, indicating that gametic imprinting is not key to the phenomenon. The direction of silencing is also not random; instead, the same progenitor species' rRNA genes are consistently silenced.

The mechanisms responsible for the establishment of nucleolar dominance are poorly understood but once established there is a developmental component to the maintenance of the silenced state. In hybrids within the plant genera Crepis and Triticea, underdominant NORs suppressed in vegetative tissues are active in male meiocytes [8], [9]. In Brassica allotetraploid hybrids, underdominant rRNA genes that are silenced in organs derived from the vegetative and inflorescence meristems (e.g. leaves, cauline leaves, inflorescence stem) are activated, or derepressed, in all organs derived from the floral meristem, including sepals and petals [10]. These latter organs do not give rise to germ cells indicating that meiosis is not required for underdominant NORs to be derepressed. Collectively, the Crepis, Triticale and Brassica observations indicate that maintenance of nucleolar dominance is developmentally regulated such that NORs that are inactive in vegetative organs of mature plants are derepressed upon the transition to flowering and/or gametogenesis.

**Figure 1:**
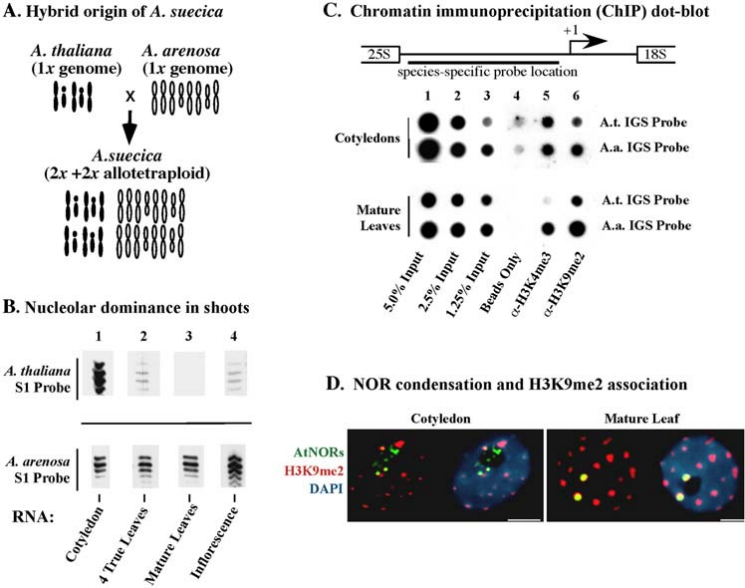
Establishment of nucleolar dominance in *Arabidopsis suecica* shoots occurs during early postembryonic development. A. Genomic composition of *A. suecica*. *A. suecica* is an allotetraploid hybrid that possesses a 2*x* chromosome complement from both *A. thaliana* (1*x* = 5 chromosomes) and *A. arenosa* (1*x* = 8 chromosomes). B. Analysis of rRNA transcripts in cotyledons and leaves. RNA isolated from cotyledons, pooled RNA from the first 4 true leaves to emerge, and fully expanded leaves of mature plants was subjected to S1 nuclease protection using probes specific for either *A. thaliana* (top row) or *A. arenosa-*derived (bottom row) rRNA transcripts. Images are from the same exposure of the same autoradiogram. C. Silencing of *A. thaliana-*derived rRNA genes is accompanied by a euchromatin to heterochromatin transition, observed as the loss of H3K4me3 association and a concomitant increased association with H3K9me2. Aliquots of total input chromatin, or chromatin immunoprecipitated with antibodies specific for H3K4me3 or H3K9me2 and then captured on protein A beads, was dot-blotted in duplicate rows. Each row was then hybridized to a radioactively labeled probe derived from the rapidly evolving intergenic spacer (IGS) region of an *A. arenosa* or *A. thaliana*-rRNA gene repeat (see diagram). No antibody was present in the “beads only” control reactions, revealing the background levels of hybridization in the absence of histone immunoprecipitation. D. Simultaneous detection of *A. thaliana* NORs (green signals) and H3K9me2 (red signals) by DNA-FISH and immunolocalization, respectively, in nuclei of cotyledons and mature leaves. DNA was counterstained with DAPI (blue signals). For each nucleus, a pair of images is shown; the image on the right includes the DAPI signal in order to reveal the position of the nucleolus, which appears as a black hole due to the paucity of nucleolar DNA. Size bars denote 5 micrometers.

DNA methylation and repressive histone modifications work in concert to silence rRNA genes subjected to nucleolar dominance. In Triticale, Brassica or Arabidopsis hybrids, treatment with chemical inhibitors of cytosine methylation (aza-dC) and/or histone deacetylation (TSA) is sufficient to derepress the silenced NORs and rRNA genes [11], [12], [13], [14], [15]. Reverse-genetic approaches using *Arabidopsis suecica*, the allotetraploid hybrid of *A. thaliana* and *A. arenosa* (see Figure 1A), have begun to identify specific chromatin modifying activities required for nucleolar dominance. In *A. suecica*, the NORs inherited from *A. thaliana* are selectively silenced whereas *A. arenosa* NORs are expressed. Using transgene-induced RNA interference (RNAi) to target the 16 predicted *Arabidopsis* histone deacetylases, we showed that *HDA6* and *HDT1* are required for nucleolar dominance. Knocking down mRNAs encoding either of these proteins is sufficient to cause the *A. thaliana*-derived rRNA genes to be expressed in fully expanded leaves of mature *A. suecica* plants [14], [16]. HDA6 deacetylates multiple lysines of multiple histones [16] and its activity is blocked by TSA, suggesting that inhibition of HDA6 activity can explain the ability of TSA to derepress the silenced *A. thaliana*-derived rRNA genes in *A. suecica* [16]. Although we have been unable to verify biochemically that HDT1 is a histone deacetylase, knocking down *HDT1* or *HDA6* by transgene-induced RNAi has similar consequences including decondensation of *A. thaliana* derived NORs, decreased association of rRNA genes with histone H3 that is dimethylated on lysine 9 (H3K9me2) and increased association with histone H3 that is trimethylated on lysine 4 (H3K4me3) [14], [16]. HDT1 and HDA6 both localize to nucleoli, where rRNA gene transcription takes place, suggesting that rRNA genes may be direct targets of their action [14], [16].

In this study, we have explored how and when nucleolar dominance becomes established. Using a combination of molecular and cytological assays, we show that nucleolar dominance is not apparent in newly germinated *A. suecica* seedling cotyledons formed during embryogenesis but becomes established during early postembryonic growth in organs and tissues derived from the stem cells of both the shoot and root apical meristems. Underdominant rRNA genes are progressively silenced and increasingly associated with heterochromatic histone and DNA methylation marks as the multi-megabase NORs become increasingly condensed. RNAi-mediated knockdown of *HDT1* or *HDA6* is sufficient to prevent the developmentally programmed inactivation and condensation of *A. thaliana* NORs, indicating that these chromatin modifiers play important roles in the postembryonic establishment of nucleolar dominance.

## Results

### Nucleolar dominance is established progressively in developing shoots and roots

In newly germinated Arabidopsis seedlings, the cotyledons that developed during embryogenesis play an important role in supplying stored carbon and nitrogen to the developing plant until the first true leaves emerge and the plant becomes photosynthetically competent and self-sufficient. An S1 nuclease protection assay using species-specific probes to detect transcripts from the *A. thaliana* or *A. arenosa*-derived rRNA genes that are present in the *A. suecica* genome revealed that rRNA genes of both progenitors are actively transcribed in cotyledons (Figure 1B), showing that nucleolar dominance does not occur in these organs. By contrast, in total RNA isolated from the first four true leaves to emerge from the shoot apical meristem, transcripts from the *A. arenosa*-derived rRNA genes are abundant whereas *A. thaliana*-derived rRNA genes are detected at much lower levels (Figure 1B), indicative of incomplete nucleolar dominance. In fully expanded rosette leaves of mature plants, *A. thaliana*-derived rRNA gene transcripts are undetectable, indicating that nucleolar dominance is complete in these leaves. Following bolting and flowering, low levels of *A. thaliana*-derived transcripts are again detected in the inflorescence tissue, consistent with prior results in Brassica and Triticale showing that nucleolar dominance is incomplete upon the transition to flowering [10] or gametogenesis [9], respectively. Collectively, these results suggest that nucleolar dominance is not evident in the major organs that developed during embryogenesis (cotyledons), is established progressively in organs that develop postembryonically from the shoot apical meristem, and is incomplete, or leaky, in reproductive tissues that arise late in development.

Chromatin immunoprecipitation (ChIP) experiments revealed previously that promoter regions of active rRNA genes in *A. suecica* are associated with histone H3 that is trimethylated on lysine 4 (H3K4me3), a euchromatic mark, whereas promoters of silenced rRNA genes are associated with H3K9me2, a heterochromatic mark [14]. By using chemical or RNAi-mediated inhibition of DNA methylation or histone deacetylation to switch rRNA genes from off to on, derepression of silenced rRNA genes is accompanied by a heterochromatin to euchromatin transition that is detected as a shift from an association of rRNA gene promoters with H3K9me2 to their association with H3K4me3 [14], [16]. ChIP analyses comparing the chromatin state of rRNA genes in cotyledons versus mature plants reveal that analogous changes in chromatin modification accompany the establishment of nucleolar dominance during postembryonic development (Figure 1C). Dot-blots were generated using aliquots of input *A. suecica* chromatin, chromatin absorbed in the absence of antibodies (“beads only” control), or chromatin immunoprecipitated by antibodies specific for H3K4me3 or H3K9me2 and then captured on protein A beads. Duplicate rows of samples were then probed for the presence of *A. thaliana* or *A. arenosa* rRNA genes using radioactively-labeled DNA corresponding to the hypervariable intergenic spacer regions (see diagram in Figure 1C). These *A. thaliana* and *A. arenosa* probes are species-specific when used for high-stringency DNA blot hybridization [14], [16] or DNA-FISH (fluorescence in situ hybridization; see below) [17], [18]. The ChIP dot-blot shows that *A. thaliana* and *A. arenosa*-derived rRNA genes are similarly associated with both H3K9me2 and H3K4me3 in cotyledons, with ∼50–70% of the genes inherited from each progenitor associated with H3K4me3 and the remainder associated with the heterochromatic mark, H3K9me2. In contrast, ChIP using chromatin isolated from mature leaves shows little association of the *A. thaliana*-derived rRNA genes with H3K4me3; instead these underdominant genes are almost exclusively associated with H3K9me2, consistent with their silencing (refer to Figure 1B; see also [14], [16]). The dominant *A. arenosa*-derived rRNA genes associate with both H3K9me2 and H3K4me3, suggesting that a subset of the rRNA genes of *A. arenosa* origin remains transcriptionally active (H3K4me3-associated) in mature leaves with the remaining rRNA genes inactive [14], [16].

The transcriptional activity of *A. thaliana*-derived rRNA genes in *A. suecica* cotyledons and their H3K9me2-associated silencing in mature leaves correlates with distinct changes in NOR organization. Figure 1D (see also Tables S1 and S2) shows a comparison of cotyledon and mature leaf nuclei subjected to DNA fluorescence *in situ* hybridization (DNA-FISH) using an *A. thaliana*-specific rRNA gene probe (green signals) in conjunction with immunolocalization of H3K9me2 (red signals). In cotyledon nuclei, the two *A. thaliana*-derived NORs are decondensed such that they yield three or more (typically 4-6) signals that are partially localized within the nucleolus, which appears as a dark region in the DAPI-stained nuclei. The decondensed *A. thaliana* NORs in cotyledon nuclei do not colocalize significantly with the H3K9me2-enriched chromocenters that are composed primarily of centromeric and pericentromeric repeats and associated heterochromatin. By contrast, in mature leaves the two NORs are highly condensed, localized external to the nucleolus and coincident with chromocenters such that the overlapping red H3K9me2 and green NOR signals appear yellow in Figure 1D.

**Figure 2:**
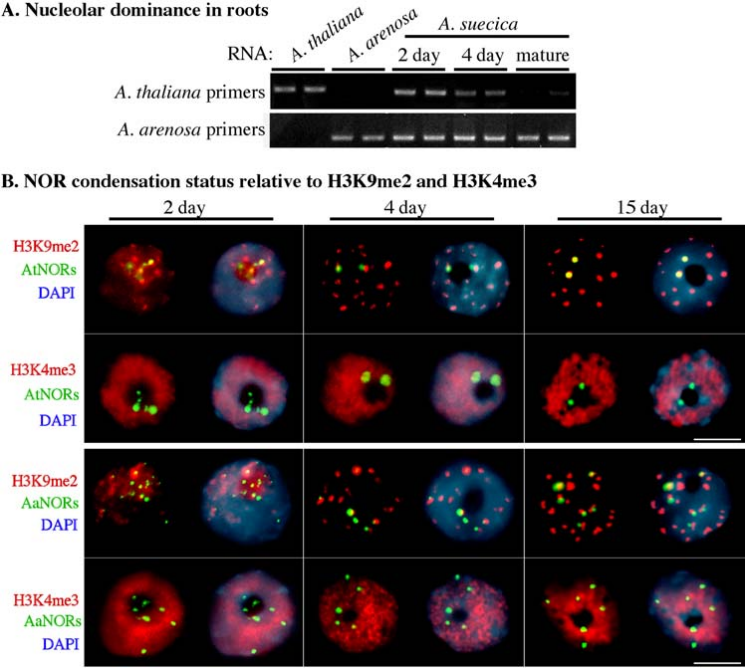
Establishment of nucleolar dominance in roots. A. Nucleolar dominance is progressively established in roots, as in leaves. Total RNA was subjected to RT-PCR using primer pairs that specifically amplify either *A. thaliana* or *A. arenosa-*derived rRNA gene transcripts. For each RNA sample, duplicate PCR reactions were conducted. The controls at the left of the figure show that *A. thaliana*-specific primers fail to yield a PCR product using *A. arenosa* RNA as the template; likewise *A. arenosa*-specific primers do not amplify *A. thaliana* RNA. Remaining reactions utilized total RNA isolated from roots harvested 2 days, 4 days and >3 weeks post-germination. B. Developmentally regulated silencing of *A. thaliana-*derived rRNA genes is accompanied by alterations in NOR organization. The degree to which NORs in *A. suecica* root tip cells are condensed and/or associated with histones bearing euchromatic (H3K4me3) or heterochromatic (H3K9me2) marks was assessed by immunolocalization of histones H3K9me2 and H3K4me3 (red signals) in combination with DNA-FISH (green signals). In the top two rows, the DNA-FISH probe is derived from the intergenic spacer of *A. thaliana* rRNA genes and is specific for *A. thaliana*-derived NORs. Likewise, in the bottom two rows an analogous probe specific for *A. arenosa*-derived NORs was used for DNA-FISH. In all nuclei, DNA was counterstained with DAPI (blue signals). For each nucleus, a pair of images is shown; the image on the right includes the DAPI signal. Size bars denote 5 micrometers.

Progressive establishment of nucleolar dominance also occurs in roots, as revealed by an RT-PCR assay that makes use of species-specific primer pairs to amplify either *A. thaliana* or *A. arenosa*-derived rRNA gene transcripts (Figure 2A). In RNA purified from root tips (∼1 cm) of seedlings harvested 2 days after germination, both the *A. thaliana* and *A. arenosa*-derived rRNA gene transcripts are readily detected in similar abundance. However, by 4 days post-germination, *A. thaliana*-derived rRNA gene transcripts are less abundant than *A. arenosa*-derived transcripts and in root tips of mature *A. suecica* plants, *A. thaliana*-derived rRNA gene transcripts are detectable in only trace amounts.

Cytological changes that correlate with the progressive silencing of *A. thaliana* rRNA genes in developing roots can be detected using intergenic spacer probes that allow *A. thaliana* and *A. arenosa*-derived rRNA genes to be discriminated by DNA-FISH (Figure 2B) [17], [18]. In root cell nuclei isolated from the meristematic zone of 15 day old plants, the two NORs of *A. thaliana* origin are highly condensed and are colocalized with H3K9me2- enriched chromocenters (Figure 2B; see Tables S3 and S4 for quantitative data), as in leaf nuclei of mature plants (compare to Figure 1D). The *A. thaliana* NORs in root-tip cells of 15 day old plants do not overlap with signals for H3K4me3, a mark of active euchromatin (Figure 2B, second row from top). Taken together, these results show that in roots the *A. thaliana* rRNA genes that are subjected to nucleolar dominance are associated with H3 that is dimethylated on lysine 9 but are not associated with H3 that is trimethylated on lysine 4, in agreement with the ChIP dot-blot data (compare data of Figures 1 and 2) and previous studies using leaf cell nuclei of mature plants [14], [16].

In root nuclei of 15 day old plants, the six NORs in *A. suecica* that are derived from *A. arenosa* partially, but imperfectly, overlap with heterochromatic chromocenters enriched for H3K9me2 but unlike *A. thaliana*-derived NORs they also overlap with the immunolocalization signals for H3K4me3 (see Tables S5 and S6 for quantitative data), which marks the active euchromatin (Figure 2B, bottom two rows). These observations are consistent with the ChIP data of Figure 1 and the interpretation that a subset of rRNA genes within *A. arenosa*-derived NORs is active, H3K4me3-associated and partially decondensed whereas the remaining, presumably excess genes, are inactive, condensed and associated with H3K9me2 [14].

Unlike the situation in plants 15 days post-germination, in which the two *A. thaliana-*derived NORs are highly condensed and transcripts from their rRNA genes are barely detectable, the *A. thaliana*-derived NORs are significantly decondensed (Fisher's exact test: P<0.0001; see Tables S3 and S4 for quantitative data) in root cell nuclei of plants two days post-germination (Figure 2B). In these nuclei, three or more DNA-FISH signals (typically 4-6) are observed using the *A. thaliana*-specific probe, indicative of condensed portions of the NORs that are interspersed with decondensed strings of active rRNA genes whose chromatin fibers are too thin to be observed directly. The decondensed *A. thaliana*-derived NORs in two day old roots partially overlap with H3K9me2 foci, which are not yet as condensed or as prominent as in nuclei 4 or 15 days post-germination. The age-dependent differences in NOR-H3K9me2 association in 2, 4 and 15 day old plants is statistically significant (P<0.0001). The *A. thaliana*-derived NORs in 2 day old plants partially overlap with H3K4me3 signals, unlike 15 day-old plants (P<0.0001), consistent with the expression of *A. thaliana*-derived rRNA genes detected 2 days post-germination (refer to Figure 2A).

Nucleolar dominance is not yet established in roots 4 days post-germination (Figure 2A). Cytologically, the *A. thaliana-*derived NORs in root tip cells 4 days post-germination typically appear as two large foci that are diffuse in appearance and are not as condensed as in nuclei of 15 day old plants (P<0.0001). These NORs substantially overlap the H3K9me2-enriched chromocenters, which are more organized than in root-tip nuclei 2 days post-germination, but also continue to overlap H3K4me3 signals (see Tables S3 and S4 for quantitative data). Therefore, the cytological phenotype of the *A. thaliana*-derived NORs at 4 days is intermediate between the 2 day and 15 day-old phenotypes (Figure 2A).

**Figure 3:**
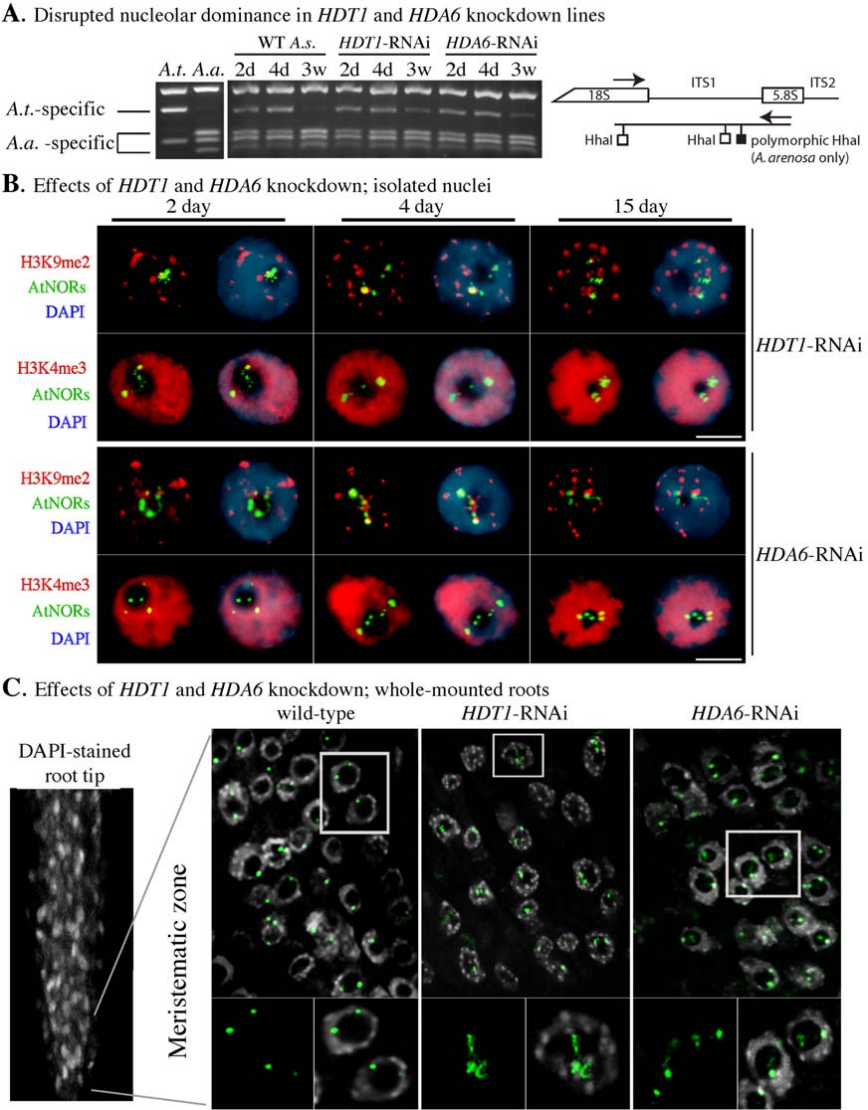
Histone deacetylases HDT1 and HDA6 are required for *A. thaliana* NOR condensation and heterochromatinization during early postembryonic development. A. Establishment of nucleolar dominance is impaired in *HDT1*-RNAi and *HDA6*-RNAi lines. RNA was isolated from *A. thaliana* (*A.t.*) and *A. arenosa* (*A.a.*) controls, as well as from wild-type (non-transgenic) and T3 generation *HDT1*- and *HDA6*-RNAi *A. suecica* lines. Following RT-PCR using primers that flank internal transcribed spacer 1 (ITS1), amplified cDNA was subjected to *Hha*I digestion, which allows A.t. and A.a.-derived rRNA gene transcripts to be discriminated following agarose gel electrophoresis (see diagram). B. Combined histone immunolocalization (red signals) and DNA-FISH detection of *A. thaliana*-derived NORs (green signals) in root nuclei of *A. suecica HDT1*-RNAi and *HDA6*-RNAi lines. DNA was counterstained with DAPI. The size bars denote 5 micrometers. C. DNA-FISH detection of *A. thaliana*-derived NORs (green signals) in the meristematic zone of whole-mounted root tips 15 days post-germination. DNA was counterstained with DAPI (grey signals). The image at left shows a stained root tip for which the position of the meristematic zone is indicated. The meristematic zone nuclei of wild-type, HDT1-RNAi and HDA6-RNAi plants that are enclosed by rectangles are shown at higher magnification, with and without the DAPI signals, in the bottom row of images.

Collectively, the cytogenetic and molecular data of Figures 1 and 2 indicate that the establishment of nucleolar dominance occurs postembryonically in tissues derived from the shoot and root apical meristems, with the progressive silencing of *A. thaliana*-derived rRNA genes correlating with the progressive condensation and heterochromatinization of *A. thaliana*-derived NORs. Confocal microscopic analyses of DNA-FISH signals in whole-mounted root tips reveal that adjacent cells throughout the meristematic zones display NOR condensation phenotypes similar to those of the isolated nuclei shown in Figure 2B (Figure S1; see also Figure 3C). Therefore, the changes in NOR phenotype appear to reflect changes that are primarily dependent on the age of the plant and secondarily affected by the position of the cells relative to the stem cells at the root tip.

### *A. thaliana*-derived NORs fail to condense in *HDA6* and *HDT1* knockdown lines

We showed previously that nucleolar dominance is disrupted in leaves of mature *A. suecica* plants in which the *HDA6* or *HDT1* genes have been knocked down using transgene-induced RNA interference (RNAi) [14], [16]. To verify that nucleolar dominance continues to be disrupted in T3 generation plants descended from the *HDA6-* and *HDT1*-RNAi plants characterized in our previous studies, we performed RT-PCR of an interval spanning ITS1 (internal transcribed spacer 1) followed by digestion with the restriction endonuclease *Hha*I. In this assay, *A. thaliana* and *A. arenosa* pre-rRNA transcripts can be discriminated from one another due to a single nucleotide polymorphism that generates an additional *Hha*I site in the *A. arenosa* rRNA genes (see A.t. and A.a. controls of Figure 3A and the accompanying diagram). As shown in Figure 3A, expression of *A. thaliana*-derived rRNA genes is apparent in roots of 2 day, 4 day and 3 week-old HDT1-RNAi or HDA6-RNAi T3 plants whereas transcription of the *A. thaliana* rRNA genes is almost completely extinguished in 3 week-old wild-type plants. RT-PCR experiments also confirmed that HDT1 and HDA6 mRNA levels were knocked down several fold in the T3 RNAi lines relative to non-transgenic plants, but were not eliminated (data not shown).

To examine the cytological consequences of HDT1 and HDA6 knockdown, we examined interphase root cell nuclei of *HDT1*-RNAi and *HDA6*-RNAi plants at 2, 4 and 15 days post-germination (Figure 3; for quantitative data see Tables S3 and S4). In wild-type cells of 15 day-old *A. suecica* plants, recall that *A. thaliana*-derived NORs are observed as two highly condensed foci that are external to the nucleolus and are excluded from the H3K4me3-enriched euchromatic space, colocalizing instead with H3K9me2-enriched chromocenters (refer to Figure 2B). By contrast, the *A. thaliana*-derived NORs in *HDT1-*RNAi and *HDA6-*RNAi lines have a significantly different organization (P<0.0001), remaining decondensed throughout postembryonic development such that they are detected by DNA-FISH as three or more (typically 4-6) irregularly shaped foci (Figure 3A). These DNA-FISH foci are condensed sub-domains of the NORs that are interspersed with, and interconnected by, decondensed chromatin fibers that are below the limits of detection, as discussed previously.

Confocal microscopic analysis of nuclei within the meristematic zone of whole-mounted primary root tips 15 days post-germination (Figure 3B) shows that nuclei of cells throughout this region display NOR condensation phenotypes that are similar to the phenotypes defined using isolated nuclei (Figure 3A). Moreover, NOR condensation phenotypes in meristematic zones of lateral roots, which are initiated from secondary meristems, are indistinguishable from the corresponding phenotypes observed in primary roots derived from the root apical meristem in both wild-type and *HDA6*-RNAi plants (Figure 3B and Figure S2).

Throughout the 15 day period of root development examined cytologically, portions of the *A. thaliana*-derived NOR FISH signals in *HDT1*-RNAi and *HDA6*-RNAi plants colocalize with H3K4me3 within the euchromatic space, or within the nucleolus where rRNA gene transcription takes place (Figure 3A), whereas NORs in cells of 15 day old wild-type plants are excluded from the euchromatic space (Figure 2B; Table S4). *A. thaliana*-derived NORs in 15 day-old *HDT1*-RNAi and *HDA6*-RNAi plants also fail to colocalize with H3K9me2, unlike wild-type plants (P<0.0001). Collectively, the molecular and cytological observations indicate that HDT1 and HDA6 are required for the establishment of nucleolar dominance and the heterochromatinization of underdominant NORs during early development.

### Changes in HDT1 and HDA6 localization during development

**Figure 4:**
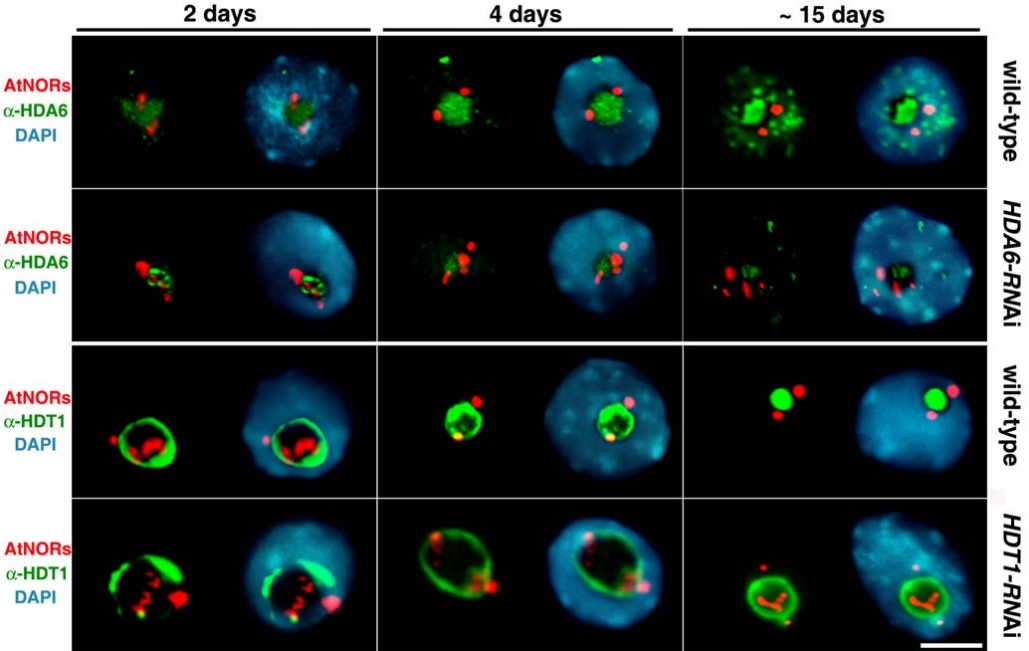
HDT1 and HDA6 nuclear distributions at different developmental stages in *A. suecica* roots. Antibodies specific for HDA6 or HDT1 were used to immunolocalize the proteins (green signals) relative to *A. thaliana*-derived NORs detected by DNA-FISH (red signals). DNA was counterstained with DAPI. The size bar denotes 5 micrometers.

Both HDT1 and HDA6 have been shown to localize to the nucleolus in leaf cells of mature plants, with HDT1 localized exclusively within the nucleolus and HDA6 localized in the nucleolus as well as the nucleoplasm [14], [16]. We questioned whether HDA6 or HDT1 localization is developmentally regulated by examining the subcellular localization of HDA6 and HDT1 relative to the *A. thaliana*-derived NORs in root-tip nuclei 2, 4 and 15 days post-germination (Figure 4; Tables S7 and S8). In wild-type *A. suecica* (lab strain LC1), HDA6 (green signals) is primarily detected in the nucleolus of root cell nuclei at 2 and 4 days post-germination. As a control, HDA6 is also detected in the nucleolus of *A. thaliana* cells, but little signal is observed in an *hda6* mutant of *A. thaliana* (Figure S4), indicating that the immunolocalization signals are due to HDA6 and the antibody is specific. By 15 days post-germination in *A. suecica* roots, a substantial amount of HDA6 is present outside the nucleolus, where it is detected in nucleoplasmic foci; this localization pattern closely resembles the pattern we observed previously for epitope-tagged recombinant HDA6 in nuclei of mature leaves [16]. Comparison of the HDA6 localization patterns relative to the condensed portions of the *A. thaliana*-derived NORs (red signals) suggests that the *A. thaliana*-derived rRNA genes are in closest proximity to the pool of HDA6 early in development when a significant portion of the NOR signals are present inside or along the inner periphery of the nucleolus.

HDT1 undergoes interesting changes in its localization during early development (Figure 4, third row; Table S7). In nuclei of root cells two days post-germination, a time at which both *A. thaliana* and *A. arenosa*-derived rRNA genes are active, HDT1 localizes primarily as a ring around the inner periphery of the nucleolus. This ring-like localization pattern was confirmed by analysis of optical sections obtained using confocal microscopy (Figure S3). At 15 days post-germination, the HDT1 signal is more uniformly distributed throughout the nucleolus (Figure 4 and Figure S3); meanwhile, the *A. thaliana*-derived rRNA genes become increasingly condensed and segregated to the outer periphery of the nucleolus. At present we lack an *hdt1* null mutant in which the specificity of the antibody can be tested by comparing immunolocalization signals in mutant and wild-type cells, however a YFP-HDT1 fusion protein also localizes to the nucleolus (Figure S5), suggesting that the immunolocalization data are reliable.

In *HDA6*- and *HDT1*-RNAi lines, the immunolocalization signals for both proteins are reduced in intensity (Figure 4), but not absent, consistent with the fact that RNAi reduces but does not eliminate expression of targeted mRNAs. Based on the signal intensities, we estimate that HDT1 may only be knocked down approximately two-fold whereas HDA6 is knocked down five-fold or more. It is intriguing that in the *HDA6*- and *HDT1*-RNAi lines the nuclei at 2, 4 and 15 days post-germination tend to display the NOR organizations typical of 2 day-old wild-type plants. Specifically, in the RNAi lines, the *A. thaliana*-derived NORs remain partially decondensed and localized internal to the nucleolus throughout development. Moreover, the HDT1 localization pattern tends to persist as a ring around the inner periphery of the nucleolus in HDT1-RNAi lines, as in 2 day old nuclei of wild-type plants (for quantitative data, see Table S7).

### *HDA6* and *HDT1* knockdown affects NOR colocalization with 5-methylcytosine-enriched foci

**Figure 5:**
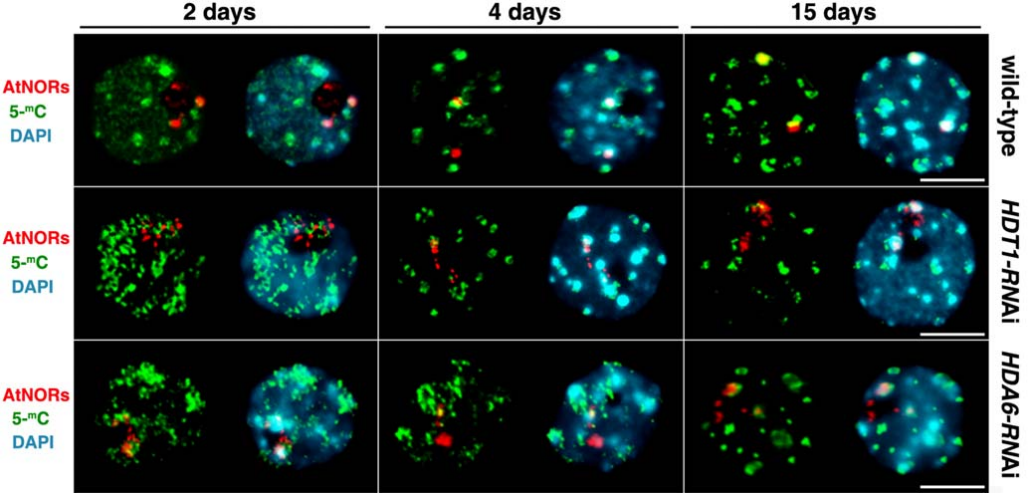
Colocalization of *A. thaliana* NORs with 5-methylcytosine-enriched chromocenters is disrupted in *HDT1-* and *HDA6*-RNAi lines. *A. suecica* root nuclei isolated from 2, 4 and 15 day-old plants was subjected to simultaneous immunolocalization of 5-methylcytosine (green signals) and DNA-FISH detection of *A. thaliana*-derived NORs (red signals). DNA was counterstained with DAPI (blue signals). The size bars denote 5 micrometers. Note the nearly perfect colocalization of *A. thaliana*-derived NORs with 5-methylcytosine-enriched chromocenters in nuclei of 15 day-old wild-type roots; this fails to occur in the knockdown lines.

Effects of HDA6 and HDT1 on the progressive condensation and inactivation of *A. thaliana*-derived NORs in *A. suecica* can also be inferred from analyses of the distribution of methylated DNA (Figure 5; Table S9). In nuclei of 2 day old roots, chromocenter formation is incomplete [19], [20], [21], such that antibodies recognizing 5-methylcytosine, a molecular feature of heterochromatin [2], [22], reveal scores of punctate signals distributed widely throughout the nucleoplasm (green signals). Some of these signals partially overlap with *A. thaliana* NOR signals visualized by DNA-FISH. As development proceeds in wild-type plants, the heterochromatin coalesces into distinct chromocenters that stain intensively with DAPI (blue signals) and are enriched for 5-methylcytosine. The highly condensed *A. thaliana*-derived NORs in wild-type plants 15 days post-germination correspond to two of these prominent DAPI and 5-methylcytosine-enriched foci. However, in *HDT1*-RNAi (middle row) and *HDA6*-RNAi lines (bottom row), the *A. thaliana*-derived NORs remain decondensed and do not colocalize substantially with major 5-methylcytosine foci, unlike wild-type plants (P<0.0001), consistent with the disruption of *A. thaliana*-derived NOR heterochromatinization and nucleolar dominance in the RNAi lines.

## Discussion

Our results show that nucleolar dominance is established during early postembryonic development in *Arabidopsis suecica* and needs to be re-established in every generation. As nucleolar dominance becomes established, *A. thaliana*-derived NORs become progressively condensed and their cognate rRNA genes become increasingly associated with H3K9me2 and less associated with H3K4me3, a mark of active euchromatin. Previously, we reported the loss of NOR decondensation and the loss of repressive heterochromatic histone modifications upon treatment of *A. suecica* with chemical inhibitors of DNA methylation (aza-dC) or histone deacetylation (TSA) or upon genetic knockdown of HDT1 or HDA6 [14], [16]. Our new results show that the changes in NOR condensation and chromatin modification that we previously ascribed to active or silenced rRNA genes based on chemical treatments or gene knockdown results also apply to the changes in rRNA gene expression that accompany normal development.

Based on our previous studies in which we showed that nucleolar dominance was disrupted in mature leaves upon RNAi-mediated knockdown of either HDT1 or HDA6, it was unclear whether these activities play a role in the establishment of nucleolar dominance or its enforcement (maintenance) during late development. Our new results indicate that both HDT1 and HDA6 are required for establishment of nucleolar dominance during early vegetative development. In HDT1 and HDA6-RNAi knockdown lines, the *A. thaliana*-derived NORs of *A. suecica* do not condense or undergo the euchromatic to heterochromatic transition to the same extent as in wild-type, non-transformed plants. Both HDT1 and HDA6 localize to the nucleolus [14], [16], suggesting that these proteins might interact directly with ribosomal RNA genes, as opposed to acting indirectly through the activity of one or more trans-acting factors located elsewhere in the nucleus. At present, the biochemical activity of HDT1 is unclear, as we have been unable to detect histone deacetylase activity for HDT1 overexpressed in *E. coli* or for affinity purified epitope-tagged HDT1 expressed in transgenic plants. However, we have shown that HDA6 is a broad specificity histone deacetylase capable of removing acetyl groups from multiple lysines of multiple histones [16], suggesting that HDA6 might erase histone acetylation throughout the regions to which it is recruited. Erasure of histone acetylation from ribosomal RNA genes undergoing silencing is likely to play an important role in the heterochromatinization and condensation of the *A. thaliana*-derived NORs in *A. suecica*. Identification of the precise regions of the rRNA genes that are targeted by HDA6 and definition of the mechanisms that recruit and regulate HDA6 are priorities for future studies.

Among species closely related enough to interbreed, it is unlikely that ribosomes assembled using rRNAs encoded by the alternative genomes would differ enough (if at all) to impact the fitness of their hybrids. Instead, current thinking is that nucleolar dominance is a manifestation of the system that seems to operate in all eukaryotes, including non-hybrids, to regulate the number of active rRNA genes according to the physiological demands for ribosomes and protein synthesis [5], [14], [23], [24], [25]. Although this hypothesis does not explain why one parental set of rRNA genes should be more susceptible to complete silencing than the other, it provides a rationale for why nucleolar dominance should occur, namely as one aspect of rRNA gene dosage control.

If nucleolar dominance is indeed a reflection of rRNA gene dosage control, the lack of nucleolar dominance at specific stages of development may be an indication that both parental sets of rRNA genes are needed at these times in order to produce sufficient numbers of ribosomes to support high levels of protein synthesis. During Arabidopsis embryogenesis, large quantities of storage proteins are produced and sequestered within protein bodies in cells of the cotyledons and throughout the embryonic axis [26]. The storage proteins are then hydrolyzed into amino acids that are used for the synthesis of new proteins during germination and early seedling growth [27]. Ribosomal RNA genes of both progenitors may be needed to supply large numbers of ribosomes during these developmental stages in *A. suecica*, providing a plausible explanation for the lack of nucleolar dominance in seedlings during the first few days post-germination. By contrast, the need for ribosomes and ribosomal RNA gene transcription may not be as great in cells of roots and leaves later in development when nucleolar dominance is apparent. Similar developmentally regulated changes in rRNA gene demand are apparent in other species, such as the frog *Xenopus laevis*. In Xenopus, the 48S rRNA genes are amplified more than 5000-fold in oocytes, to a final number of nearly 2 million [28], in order to supply enough rRNA for the transcriptionally-inactive eggs and early embryos to progress to the mid-blastula transition (∼8,000 cells) stage when transcription by RNA polymerases I, II and III resumes [29]. Likewise, the approximately 20,400 genes encoding 5S rRNA are all expressed in the oocytes but only ∼400 remain active in somatic cells [30]. By analogy, plants may also have specific times in development during which the need for ribosomal RNA genes far exceeds the needs at other times of development.

Within a root tip at a given age post-germination, the degree of NOR condensation/decondensation is similar as one moves from the region occupied by the stem cells to the adjacent region in which the daughter cells proliferate; collectively, these regions comprise the meristematic zone. Our analyses reveal that the NOR condensation phenotype within the root tip meristematic zones differs in an age-dependent manner. It is tempting to speculate that age-dependent changes in NOR condensation and nucleolar dominance are manifestations of epigenetic changes in the stem cells that are then propagated among daughter cells through multiple rounds of mitosis. Similar changes in meristematic gene activity are presumably responsible for the phenomenon of phase change in the shoot apical meristem whereby the meristem initially gives rise to leaves and shoots that display juvenile traits but later gives rise to leaves and shoots that display adult traits [31]. By genetically manipulating the epigenetic regulatory potential of meristematic stem cells it may ultimately be possible to test this hypothesis.

## Methods

### Plant Material

Seeds of *A. suecica* wild-type (Pikaard lab strain LC1), *HDT1-RNAi* [14] or *HDA6-RNAi* [16] lines were grown on sterile Murashige-Skoog germination medium in a growth chamber. The *HDT1*-RNAi and *HDA6*-RNAi plants were T3 generation descendants of transgenic plants described in previous studies [14], [16] at the T1 and T2 generations. T3 lines in which all tested individuals of the family showed stable knockdown phenotypes were used in this study. Plantlets were collected at 2-, 4- and 15 days post-germination, with germination defined as the time of primary root emergence.

### Detection of rRNA transcripts

S1 nuclease protection using 5′ end-labeled *A. thaliana* or *A. arenosa-*specific promoter probes was as described previously [14]. Briefly, probes and RNA were hybridized overnight at 50°C and resulting probe-RNA hybrids were digested with 750 units/ml S1 nuclease (Invitrogen) at 50°C for 45 min. Resulting digestion products were resolved on a sequencing gel, dried onto filter paper and exposed to X-ray film. For detection of *A. thaliana* or *A. arenosa*-derived rRNA gene transcripts by RT-PCR in Figure 2A, 500 ng of total RNA was treated with DNase RQ1 and reverse transcribed using gene specific primers and SSII reverse transcriptase (Invitrogen). cDNA was amplified using *A. thaliana*-specific rRNA gene primers 5′-ACGGGTGGCAAAGATTTCG-3′ and 5′-ATGGTTCCGCGACTCCTCC-3′ or *A. arenosa*-specific rRNA gene primers 5′-GGTTGTTACGTCGTGCGG-3′ and 5′-CGAATATCGGAGCCGCAG-3′. The RT-PCR-CAPS assay of Figure 3A was performed as previously described [16], [32].

### Chromatin immunoprecipitation

Chromatin immunoprecipitation was as described previously [14]. Briefly, nuclei of seedlings crosslinked in 1% formaldehyde were sonicated, and soluble chromatin was subjected to immunoprecipitation using antibodies specific for H3K9me2 or H3K4me3 (Upstate Cell Signaling Solutions). Chromatin-antibody complexes were captured on protein A-agarose beads, washed, then eluted with 1% SDS, 0.1M NaHC0_3_. DNA-protein crosslinks were reversed at 65°C overnight. Purified DNA was dot blotted onto Genescreen Plus membranes (Perkin-Elmer) and hybridized to labeled intergenic spacer probes as described previously [14].

### Antibody production and affinity purification

Anti-HDT1 and HDA6 antibodies were raised against recombinant proteins expressed in *E. coli*. Briefly, *HDT1* and *HDA6* full-length cDNA coding regions cloned in Gateway-compatible pENTR (Invitrogen) vectors [33] were recombined into pDEST17 (Invitrogen) to add an N-terminal 6× His tag. Resulting plasmids were transformed into BL21-AI cells grown at 37°C in a shaker-incubator. When cultures reached an A_600_ of 0.5-1.0, protein expression was induced by addition of 0.2% (w/v) L-arabinose and cultures were incubated an additional 12–16 hrs at 16°C. Cells collected by centrifugation at 5,000×g, 15 min, 4°C, were resuspended in 10 ml of 10 mM Tris-HCl pH 7.6, 500 mM NaCl, 5 mM imidazole, sonicated to lyse the cells and centrifuged at 12,000×g, 15 min, 4°C. Soluble proteins were purified by nickel-agarose chromatography (Novagen). Proteins were eluted using 10 mM Tris-HCl (pH 8.0), 300 mM NaCl and 250 mM imidazole. Approximately 2 mg of purified recombinant HDA6 and HDT1 were then subjected to SDS-PAGE and excised from the gel. Gel slices were submitted to Cocalico Biologicals, Inc. for antibody production in rabbits.

To affinity purify anti-HDT1 and anti-HDA6 antibodies, 0.5 mg of purified HDT1 and HDA6 proteins were subjected to electrophoresis on a 12.5% SDS-PAGE gel and electroblotted to PVDF membranes. PVDF strips containing the HDT1 or HDA6 protein bands, visualized by staining with Coomassie Blue, were destained using 100% methanol and washed in TBST buffer (50 mM Tris-HCl, 138 mM NaCl, 2.7 mM KCl, 0.05% Tween-20, pH 8.0), 30 minutes. 1.0 ml of antisera mixed with 9 ml of TBST was then incubated with the HDT1 or HDA6 membrane strips at 4°C, 1 hour. Membranes were washed twice with 10 ml TBST. Bound antibodies were eluted from the PVDF membranes in 0.5 M glycine, 150 mM NaCl (pH 2.5). 1M Tris-HCl, pH 8 was added to a final volume of 10% (v/v) to neutralize the pH. Aliquots were frozen in liquid nitrogen and stored at −80°C.

### DNA-FISH and protein immunolocalization in isolated nuclei

For immunodetection of modified histones, HDT1 or HDA6, root meristem nuclei were isolated and fixed in 4% paraformaldehyde in Phosphate Buffered Saline (PBS) as described previously [14]. Nuclei were incubated overnight at 4°C with the following polyclonal antibodies: anti-Histone H3 dimethyl K9 (H3K9me2, Abcam, 1∶200 working dilution), anti-Histone H3 trimethyl K4 (H3K4me3, Abcam, 1:800 dilution), anti-HDT1 (1∶400) or anti-HDA6 (1∶50). Protein-antibody complexes were detected using anti-rabbit secondary antibodies coupled to Rhodamine or Fluorescein (1:200; Sigma-Aldrich). DNA fluorescence *in situ* hybridization (DNA-FISH) was performed following post-fixation in 4% paraformaldehyde (in PBS).

For immunodetection of 5-methylcytosine in combination with DNA-FISH, root meristems were fixed in ethanol:acetic acid (3∶1) and isolated as described previously (Pontes et al., 2003). Slides were baked at 60°C, denatured in 70% formamide, 2×SSC, 50 mM sodium phosphate at 80°C for 5 min and washed in ice-cold PBS. The detection of 5-methylcytosine was performed using a monoclonal antibody against 5-methylcytidine (1∶100 dilution; AbD Serotec) followed by incubation with rabbit anti-mouse antibody conjugated to Alexa 488 (1:200 dilution, Molecular Probes) as the secondary antibody. Slides were then processed for DNA-FISH.

*A. thaliana* and *A. arenosa* species-specific NOR probes were generated by nick translation of cloned rRNA gene intergenic regions in the presence of digoxigenin–dUTP or biotin–dUTP as described previously [17]. Hybridization conditions, post-hybridization washes and detection of FISH signals were performed according to Pontes et al., 2003. Biotinylated probe DNA was detected using streptavidin-Cy3 and digoxigenin-labeled probes were detected using anti-digoxigenin-FITC (1:250, Roche). Nuclei were counterstained with DAPI (1 mg/ml) diluted in Vectashield (Vector Laboratories). Two *A. thaliana-*derived NORs and six *A. arenosa*-derived NORs are present in *A. suecica* LC1 nuclei [17], [18], therefore FISH signals that exceed these numbers were scored as evidence of NOR decondensation. Fisher's exact test was used to compare NOR decondensation frequencies for the different development stages in the plant lines used in this study.

Analysis of fluorescence signals was performed using an epifluorescence microscope Eclipse E800i equipped with a Photometrics Coolsnap ES Mono digital camera. Images were captured separately for each fluorochrome using the appropriate excitation and emission filters, merged and pseudocolored using Adobe Photoshop 6.0 (Adobe Systems) software.

### Immunolocalization and DNA in situ hybridization in whole-mounted root segments

Root tips of *A. suecica* LC1 seedlings were fixed in 4% paraformadeheyde in PBS for 30 min and washed extensively in PBS to remove the fixative. Roots used for DNA-FISH were additionally rinsed with methanol and ethanol. After rehydration in PBS, cell walls were digested 0.5–1 h with 2% driselase in PBS at 37°C. Immunolocalization and DNA-FISH were as described above.

DNA-FISH images in whole-mount roots were acquired using a MRC-600 confocal scanning laser microscope (Bio-Rad) and processed using Image J software version 1.37 (National Institutes of Heath, USA, http://rsb.info.nih.gov/ij/). Whole-mount immunolocalization image stacks were collected with a Multiphoton Zeiss LSM 510 Meta microscope, using a 40× objective. Simultaneous scanning of FITC and DAPI signals was performed using 488-nm Ar and a 715- or 750-nm multiphoton Ti-Sapphire laser. Individual optical sections and 3D projections were analyzed using Imaris 4.1 software from Bitplane (www.bitplane.com).

## Supporting Information

Table S1 Frequencies (%) of DNA-FISH signals for A. thaliana-derived NORs in interphase nuclei of wild-type A. suecica (strain LC1) cotyledons and mature leaves
(0.03 MB DOC)

Table S2 Frequencies (%) at which H3K9me2 colocalizes with A. thaliana-derived NORs in interphase nuclei of wild-type A. suecica (strain LC1) cotyledons and mature leaves
(0.03 MB DOC)

Table S3 Frequencies (%) of DNA-FISH signals for A. thaliana-derived NORs in root tip interphase nuclei of A. suecica. Nuclei of wild-type (LC1), HDT1-RNAi and HDA6-RNAi plants were compared at 2, 4 and 15 days post-germination.
(0.04 MB DOC)

Table S4 Frequencies (%) of H3K9me2 and H3K4me3 localization patterns, relative to A. thaliana-derived NORs, in root tip interphase nuclei of A. suecica. Nuclei of wild-type (LC1), HDT1-RNAi and HDA6-RNAi plants were compared at 2, 4 and 15 days post-germination.
(0.05 MB DOC)

Table S5 Frequencies (%) of DNA-FISH signals for A. arenosa-derived NORs in root tip interphase nuclei of A. suecica. Nuclei of wild-type (LC1), HDT1-RNAi and HDA6-RNAi plants were compared at 2, 4 and 15 days post-germination.
(0.03 MB DOC)

Table S6 Frequencies (%) at which H3K9me2 and H3K4me3 colocalize with A. arenosa-derived NORs in root tip interphase nuclei of A. suecica. Nuclei of wild-type (LC1) plants were observed at 2, 4 and 15 days post-germination.
(0.05 MB DOC)

Table S7 Frequencies (%) of root meristem nuclei observed with distinct HDT1 interphase localization patterns in A. suecica. Nuclei of wild-type (lab strain LC1), HDT1-RNAi and HDA6-RNAi plants were compared at 2, 4 and 15 days post-germination.
(0.04 MB DOC)

Table S8 Frequencies (%) of root meristem nuclei observed with distinct HDA6 interphase localization patterns in A. suecica. Nuclei of wild-type (lab strain LC1), HDT1-RNAi and HDA6-RNAi plants were compared at 2, 4 and 15 days post-germination.
(0.04 MB DOC)

Table S9 Frequencies (%) of 5-methylcytosine (5-mC) localization patterns, relative to A. thaliana-derived NORs, in root tip interphase nuclei of A. suecica. Nuclei of wild-type (LC1), HDT1-RNAi and HDA6-RNAi plants were compared at 2, 4 and 15 days post-germination.
(0.04 MB DOC)

Figure S1 DNA-FISH detection of A. thaliana-derived NORs (green signals) in meristematic zone cell nuclei of whole-mounted A. suecica primary root tips. DNA was counterstained with DAPI (grey/white signals). The size bars correspond to 5 μm.
(2.93 MB TIF)

Figure S2 DNA-FISH detection of A. thaliana-derived NORs (green signals) in meristematic zone nuclei of whole-mounted A. suecica lateral root tips, comparing wild-type (strain LC1) and HDA6-RNAi plants. DNA was counterstained with DAPI (grey/white signals). The nuclei enclosed by rectangles are shown enlarged in the insets. The upper inset shows the DNA-FISH signal alone and the bottom inset includes the DAPI signals. The size bars correspond to 5 μm.
(4.38 MB TIF)

Figure S3 HDT1 immunolocalization patterns in meristematic zone nuclei of whole-mounted 2 and 15 day-old plant root tips. Anti-HDT1 antibody (α-HDT1) signals are in green; DNA counterstained with DAPI is in grey. Each panel shows a single confocal optical section that passes through the centers of the nucleoli (Nuc) in two or more neighboring cells. The nucleoli are the dark regions of the nuclei not stained by DAPI. The size bars correspond to 5 μm.
(3.80 MB TIF)

Figure S4 Immunolocalization of HDA6 protein (green signals) in wild-type (Col-0 ecotype) and hda6 (allele axe1-5 allele) mutant A. thaliana. DNA was counterstained with DAPI (blue). The size bar correspond to 5 μm. The severely reduced HDA6 signal in the axe1-5 mutant indicates that the strong signals detected in wild-type nuclei are attributable to HDA6. Moreover, epitope-tagged HDA6 expressed from a transgene and detected by virtue of its epitope tag displays the same localization pattern shown here (see Earley et al., 2006).
(0.54 MB TIF)

Figure S5 HDT1-YFP localizes to the nucleolus. An intact root of a transgenic A. thaliana plant expressing an HDT1-YFP translational fusion protein was stained with DAPI and subjected to differential interference contrast (DIC) and fluorescence microscopy. The three panels show the nucleus of a single root cell. The nucleolus is readily apparent in the DIC image and corresponds to the region of the nucleus least stained by DAPI. The YFP signal is exclusively localized within the nucleolus, as reported previously (Lawrence et al., 2004). The nucleolar localization of the HDT1-YFP fusion protein supports the nucleolar localization of HDT1 detected using anti-HDT1 antibodies.
(2.10 MB TIF)
