# Supplementary material for: Postembryonic Establishment of Megabase-Scale Gene Silencing in Nucleolar Dominance
Source: PLoS One. 2007 Nov 7;2(11):e1157. doi: 10.1371/journal.pone.0001157 (PMC2048576; doi:10.1371/journal.pone.0001157)
Supplement: Table S1 — Frequencies (%) of DNA-FISH signals for A. thaliana-derived NORs in interphase nuclei of wild-type A. suecica (strain LC1) cotyledons and mature leaves (0.03 MB DOC) [file pone.0001157.s001.doc]

**Table S1**. Frequencies (%) of DNA-FISH signals for *A. thaliana*-derived NORs in interphase nuclei of wild-type *A. suecica* (strain LC1) cotyledons and mature leaves

|  |  | Development stage | |
| --- | --- | --- | --- |
|  |  | Cotyledons | Mature leaves |
| Number of FISH signals | 1 signal | 13 | 11 |
|  | 2 signals | 23 | 75 |
|  | ≥3 signals | 64 | 14 |
|  | # Scored nuclei | 167 | 179 |
