# Supplementary material for: Postembryonic Establishment of Megabase-Scale Gene Silencing in Nucleolar Dominance
Source: PLoS One. 2007 Nov 7;2(11):e1157. doi: 10.1371/journal.pone.0001157 (PMC2048576; doi:10.1371/journal.pone.0001157)
Supplement: Table S2 — Frequencies (%) at which H3K9me2 colocalizes with A. thaliana-derived NORs in interphase nuclei of wild-type A. suecica (strain LC1) cotyledons and mature leaves (0.03 MB DOC) [file pone.0001157.s002.doc]

**Table S2**. Frequencies (%) at which H3K9me2 colocalizes with *A. thaliana*-derived NORs in interphase nuclei of wild-type *A. suecica* (strain LC1) cotyledons and mature leaves

|  |  | Development stage | |
| --- | --- | --- | --- |
|  |  | Cotyledons | Mature leaves |
| H3K9me2 and AtNORs | Colocalized | 26 | 83 |
|  | Partially colocalized | 57 | 17 |
|  | Not colocalized | 31 | 0 |
|  | # Scored nuclei | 167 | 179 |
