# Supplementary material for: Postembryonic Establishment of Megabase-Scale Gene Silencing in Nucleolar Dominance
Source: PLoS One. 2007 Nov 7;2(11):e1157. doi: 10.1371/journal.pone.0001157 (PMC2048576; doi:10.1371/journal.pone.0001157)
Supplement: Table S3 — Frequencies (%) of DNA-FISH signals for A. thaliana-derived NORs in root tip interphase nuclei of A. suecica. Nuclei of wild-type (LC1), HDT1-RNAi and HDA6-RNAi plants were compared at 2, 4 and 15 days post-germination. (0.04 MB DOC) [file pone.0001157.s003.doc]

**Table S3**. Frequencies (%) of DNA-FISH signals for *A. thaliana*-derived NORs in root tip interphase nuclei of *A. suecica*. Nuclei of wild-type (LC1), *HDT1-RNAi* and *HDA6-RNAi* plants were compared at 2, 4 and 15 days post-germination.

|  |  | Genotype | | | | | | | | |
| --- | --- | --- | --- | --- | --- | --- | --- | --- | --- | --- |
|  |  | LC1 (wild-type) | | | *HDT1-RNAi* | | | *HDA6-RNAi* | | |
|  | | 2 day | 4 day | 15 day | 2 day | 4 day | 15 day | 2 day | 4 day | 15 day |
|  | 1 signal | 0 | 5 | 11 | 12 | 5 | 5 | 12 | 8 | 9 |
| Number of FISH signals | 2 signals | 15 | 67 | 81 | 37 | 28 | 21 | 37 | 26 | 28 |
|  | ≥3 signals | 85 | 28 | 8 | 51 | 67 | 74 | 51 | 66 | 63 |
|  | # Scored nuclei | 325 | 283 | 316 | 227 | 216 | 280 | 221 | 204 | 242 |
