# Supplementary material for: Postembryonic Establishment of Megabase-Scale Gene Silencing in Nucleolar Dominance
Source: PLoS One. 2007 Nov 7;2(11):e1157. doi: 10.1371/journal.pone.0001157 (PMC2048576; doi:10.1371/journal.pone.0001157)
Supplement: Table S4 — Frequencies (%) of H3K9me2 and H3K4me3 localization patterns, relative to A. thaliana-derived NORs, in root tip interphase nuclei of A. suecica. Nuclei of wild-type (LC1), HDT1-RNAi and HDA6-RNAi plants were compared at 2, 4 and 15 days post-germination. (0.05 MB DOC) [file pone.0001157.s004.doc]

**Table S4**. Frequencies (%) of H3K9me2 and H3K4me3 localization patterns, relative to *A. thaliana*-derived NORs, in root tip interphase nuclei of *A. suecica.* Nuclei of wild-type (LC1), *HDT1-RNAi* and *HDA6-RNAi* plants were compared at 2, 4 and 15 days post-germination.

|  |  | Genotype | | | | | | | | |
| --- | --- | --- | --- | --- | --- | --- | --- | --- | --- | --- |
|  |  | LC1 | | | *HDT1-RNAi* | | | *HDA6-RNAi* | | |
|  | | 2 day | 4 day | 15 day | 2 day | 4 day | 15 day | 2 day | 4 day | 15 day |
|  | Colocalized | 33 | 46 | 75 | 12 | 13 | 31 | 38 | 44 | 29 |
| H3K9me2 and AtNORs | Partially colocalized | 41 | 37 | 15 | 65 | 62 | 58 | 49 | 51 | 55 |
|  | Not colocalized | 26 | 17 | 10 | 23 | 25 | 11 | 13 | 5 | 16 |
|  | # Scored nuclei | 61 | 74 | 66 | 58 | 51 | 65 | 53 | 55 | 68 |
|  | Colocalized | 25 | 12 | 0 | 7 | 25 | 16 | 46 | 61 | 79 |
| H3K4me3 and AtNORs | Partially colocalized | 48 | 55 | 31 | 57 | 68 | 72 | 31 | 29 | 21 |
|  | Not colocalized | 27 | 33 | 69 | 36 | 7 | 12 | 23 | 10 | 0 |
|  | # Scored nuclei | 56 | 56 | 45 | 45 | 41 | 67 | 49 | 38 | 50 |
