# Supplementary material for: Postembryonic Establishment of Megabase-Scale Gene Silencing in Nucleolar Dominance
Source: PLoS One. 2007 Nov 7;2(11):e1157. doi: 10.1371/journal.pone.0001157 (PMC2048576; doi:10.1371/journal.pone.0001157)
Supplement: Table S5 — Frequencies (%) of DNA-FISH signals for A. arenosa-derived NORs in root tip interphase nuclei of A. suecica. Nuclei of wild-type (LC1), HDT1-RNAi and HDA6-RNAi plants were compared at 2, 4 and 15 days post-germination. (0.03 MB DOC) [file pone.0001157.s005.doc]

**Table S5**. Frequencies (%) of DNA-FISH signals for *A. arenosa*-derived NORs in root tip interphase nuclei of *A. suecica*. Nuclei of wild-type (LC1), *HDT1-RNAi* and *HDA6-RNAi* plants were compared at 2, 4 and 15 days post-germination.

|  |  | | | Development stage | | |  | | |
| --- | --- | --- | --- | --- | --- | --- | --- | --- | --- |
| Number of FISH signals | 2 day | | | 4 day | | | 15 day | | |
| ≤5  6-8  ≥9 |  | 12 |  |  | 25 |  |  | 46 |  |
|  |  | 45 |  |  | 58 |  |  | 48 |  |
|  |  | 43 |  |  | 17 |  |  | 6 |  |
| # Scored nuclei |  | 137 |  |  | 132 |  |  | 140 |  |
