# Supplementary material for: Postembryonic Establishment of Megabase-Scale Gene Silencing in Nucleolar Dominance
Source: PLoS One. 2007 Nov 7;2(11):e1157. doi: 10.1371/journal.pone.0001157 (PMC2048576; doi:10.1371/journal.pone.0001157)
Supplement: Table S6 — Frequencies (%) at which H3K9me2 and H3K4me3 colocalize with A. arenosa-derived NORs in root tip interphase nuclei of A. suecica. Nuclei of wild-type (LC1) plants were observed at 2, 4 and 15 days post-germination. (0.05 MB DOC) [file pone.0001157.s006.doc]

**Table S6**. Frequencies (%) at which H3K9me2 and H3K4me3 colocalize with *A. arenosa*-derived NORs in root tip interphase nuclei of *A. suecica.* Nuclei of wild-type (LC1) plants were observed at 2, 4 and 15 days post-germination.

|  |  | Development stage | | | | | | | | |
| --- | --- | --- | --- | --- | --- | --- | --- | --- | --- | --- |
| H3K9me2 | |  | 2 day |  |  | 4 day |  |  | 15 day |  |
| Colocalized with:  ≤2 NORs  3 NORs  ≥4 NORs | |  | 15 |  |  | 7 |  |  | 0 |  |
|  |  |  | 40 |  |  | 32 |  |  | 19 |  |
|  |  |  | 45 |  |  | 61 |  |  | 81 |  |
|  | # Scored nuclei |  | 72 |  |  | 58 |  |  | 66 |  |
| H3K4me3 | |  | 2 day |  |  | 4 day |  |  | 15 day |  |
| Colocalized with:  ≤2 NORs | |  | 7 |  |  | 9 |  |  | 0 |  |
| 3 NORs | |  | 28 |  |  | 22 |  |  | 5 |  |
| ≥4 NORs | |  | 65 |  |  | 69 |  |  | 95 |  |
|  | # Scored nuclei |  | 74 |  |  | 65 |  |  | 53 |  |
