# Supplementary material for: Postembryonic Establishment of Megabase-Scale Gene Silencing in Nucleolar Dominance
Source: PLoS One. 2007 Nov 7;2(11):e1157. doi: 10.1371/journal.pone.0001157 (PMC2048576; doi:10.1371/journal.pone.0001157)
Supplement: Table S7 — Frequencies (%) of root meristem nuclei observed with distinct HDT1 interphase localization patterns in A. suecica. Nuclei of wild-type (lab strain LC1), HDT1-RNAi and HDA6-RNAi plants were compared at 2, 4 and 15 days post-germination. (0.04 MB DOC) [file pone.0001157.s007.doc]

**Table S7**. Frequencies (%) of root meristem nuclei observed with distinct HDT1 interphase localization patterns in *A. suecica.* Nuclei of wild-type (lab strain LC1), *HDT1-RNAi* and *HDA6-RNAi* plants were compared at 2, 4 and 15 days post-germination.

|  |  | Genotype | | | | | |
| --- | --- | --- | --- | --- | --- | --- | --- |
|  |  | LC1 | | | *HDT1-RNAi* | | |
| Development stage | | 2 day | 4 day | 15 day | 2 day | 4 day | 15 day |
| HDT1 localization pattern | Throughout nucleolus | 4 | 11 | 81 | 0 | 8 | 14 |
|  | Nucleolar ring | 73 | 55 | 3 | 64 | 71 | 70 |
|  | Nucleoplasmic | 23 | 34 | 16 | 36 | 21 | 16 |
|  | Scored nuclei | 71 | 49 | 61 | 72 | 67 | 75 |
