# Supplementary material for: Postembryonic Establishment of Megabase-Scale Gene Silencing in Nucleolar Dominance
Source: PLoS One. 2007 Nov 7;2(11):e1157. doi: 10.1371/journal.pone.0001157 (PMC2048576; doi:10.1371/journal.pone.0001157)
Supplement: Table S8 — Frequencies (%) of root meristem nuclei observed with distinct HDA6 interphase localization patterns in A. suecica. Nuclei of wild-type (lab strain LC1), HDT1-RNAi and HDA6-RNAi plants were compared at 2, 4 and 15 days post-germination. (0.04 MB DOC) [file pone.0001157.s008.doc]

**Table S8**. Frequencies (%) of root meristem nuclei observed with distinct HDA6 interphase localization patterns in *A. suecica*. Nuclei of wild-type (lab strain LC1), *HDT1-RNAi* and *HDA6-RNAi* plants were compared at 2, 4 and 15 days post-germination.

|  |  | Genotype | | | | | |
| --- | --- | --- | --- | --- | --- | --- | --- |
|  |  | LC1 | | | *HDA6-RNAi* | | |
| Development stage | | 2 day | 4 day | 15 day | 2 day | 4 day | 15 day |
| HDA6 localization pattern | Nucleoplasm and nucleolus | 53* | 49* | 67 | 63* | 71* | 73* |
|  | Nucleoplasm | 17* | 11* | 26 | 27* | 18* | 14* |
|  | Nucleolus | 30* | 40* | 7 | 10* | 11* | 13* |
|  | Scored nuclei | 78 | 52 | 68 | 63 | 62 | 72 |

* Reduced signal intensity
