# Supplementary material for: Postembryonic Establishment of Megabase-Scale Gene Silencing in Nucleolar Dominance
Source: PLoS One. 2007 Nov 7;2(11):e1157. doi: 10.1371/journal.pone.0001157 (PMC2048576; doi:10.1371/journal.pone.0001157)
Supplement: Table S9 — Frequencies (%) of 5-methylcytosine (5-mC) localization patterns, relative to A. thaliana-derived NORs, in root tip interphase nuclei of A. suecica. Nuclei of wild-type (LC1), HDT1-RNAi and HDA6-RNAi plants were compared at 2, 4 and 15 days post-germination. (0.04 MB DOC) [file pone.0001157.s009.doc]

**Table S9**. Frequencies (%) of 5-methylcytosine (5-mC) localization patterns, relative to *A. thaliana*-derived NORs, in root tip interphase nuclei of *A. suecica.* Nuclei of wild-type (LC1), *HDT1-RNAi* and *HDA6-RNAi* plants were compared at 2, 4 and 15 days post-germination.

|  |  | Genotype | | | | | | | | |
| --- | --- | --- | --- | --- | --- | --- | --- | --- | --- | --- |
|  |  | LC1 | | | *HDT1-RNAi* | | | *HDA6-RNAi* | | |
|  | | 2 day | 4 day | 15 day | 2 day | 4 day | 15 day | 2 day | 4 day | 15 day |
|  | Colocalized | 31 | 25 | 39 | 7 | 2 | 15 | 21 | 35 | 23 |
| 5-mC and AtNORs | Partially colocalized | 43 | 59 | 53 | 65 | 54 | 71 | 56 | 38 | 61 |
|  | Not colocalized | 26 | 16 | 8 | 28 | 44 | 14 | 23 | 27 | 16 |
|  | # Scored nuclei | 59 | 63 | 69 | 52 | 57 | 73 | 56 | 50 | 52 |
