# Supplementary figures and images for: Postembryonic Establishment of Megabase-Scale Gene Silencing in Nucleolar Dominance
Source: PLoS One. 2007 Nov 7;2(11):e1157. doi: 10.1371/journal.pone.0001157 (PMC2048576; doi:10.1371/journal.pone.0001157)

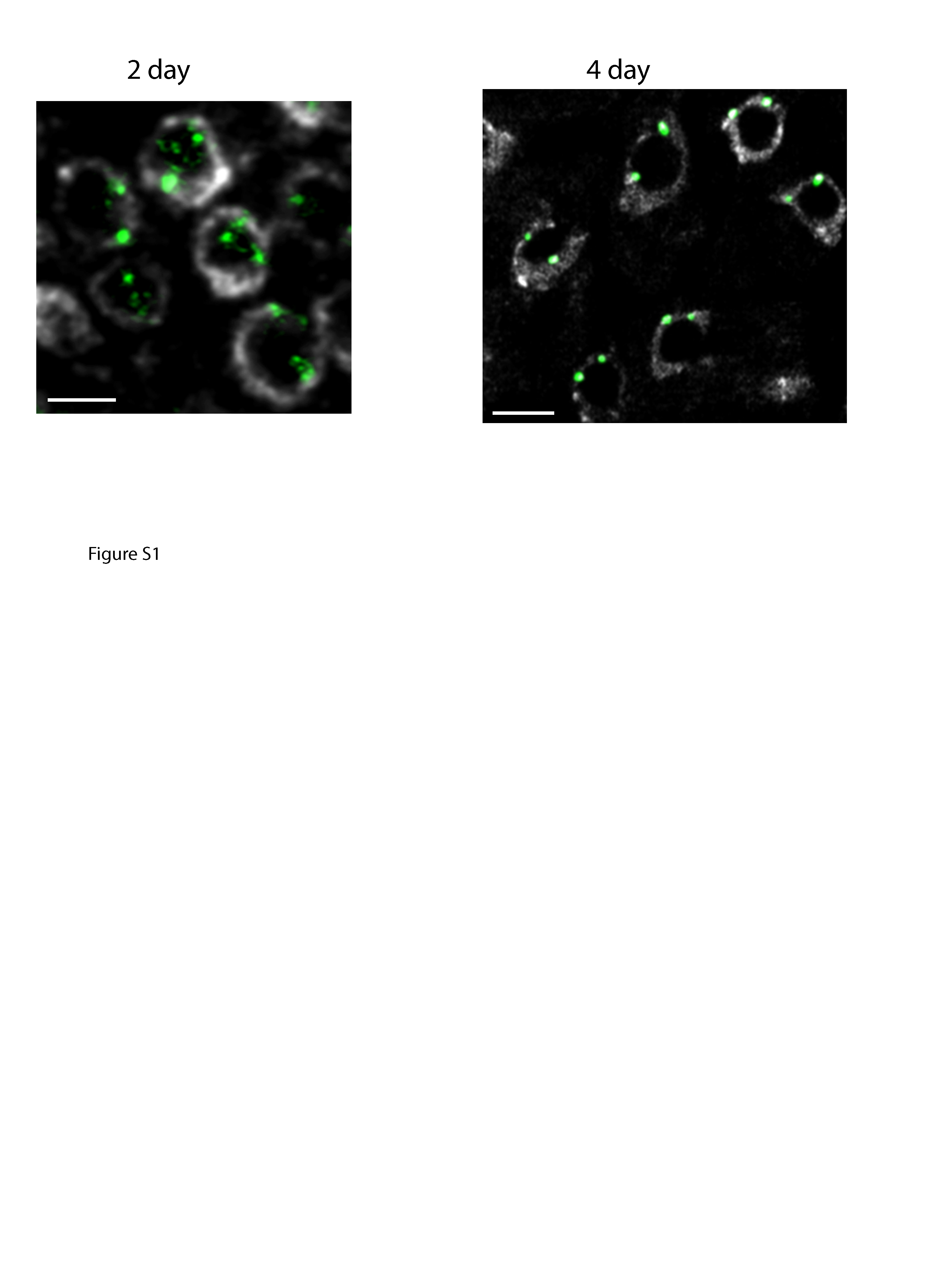

Supplement: Figure S1 — DNA-FISH detection of A. thaliana-derived NORs (green signals) in meristematic zone cell nuclei of whole-mounted A. suecica primary root tips. DNA was counterstained with DAPI (grey/white signals). The size bars correspond to 5 μm. (2.93 MB TIF) [file pone.0001157.s010.tif]

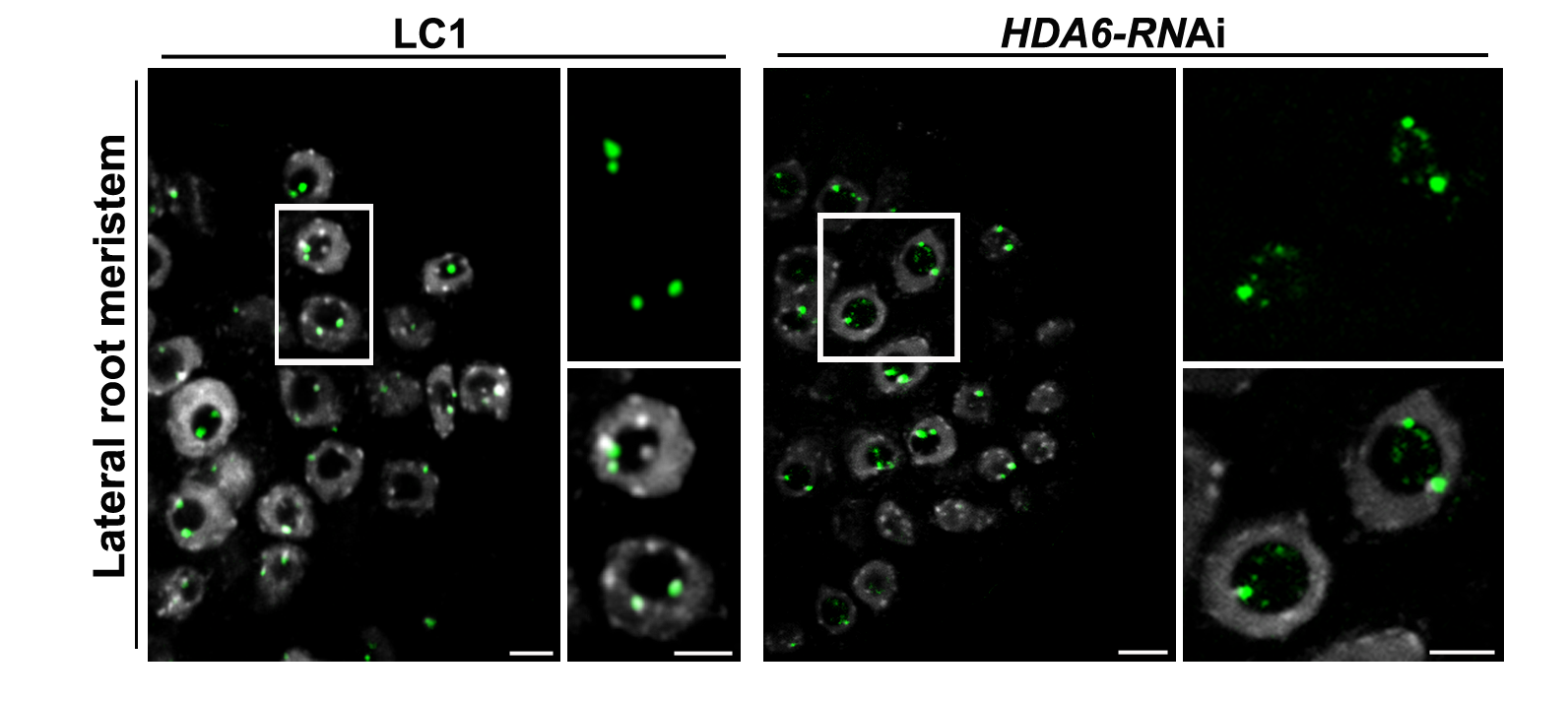

Supplement: Figure S2 — DNA-FISH detection of A. thaliana-derived NORs (green signals) in meristematic zone nuclei of whole-mounted A. suecica lateral root tips, comparing wild-type (strain LC1) and HDA6-RNAi plants. DNA was counterstained with DAPI (grey/white signals). The nuclei enclosed by rectangles are shown enlarged in the insets. The upper inset shows the DNA-FISH signal alone and the bottom inset includes the DAPI signals. The size bars correspond to 5 μm. (4.38 MB TIF) [file pone.0001157.s011.tif]

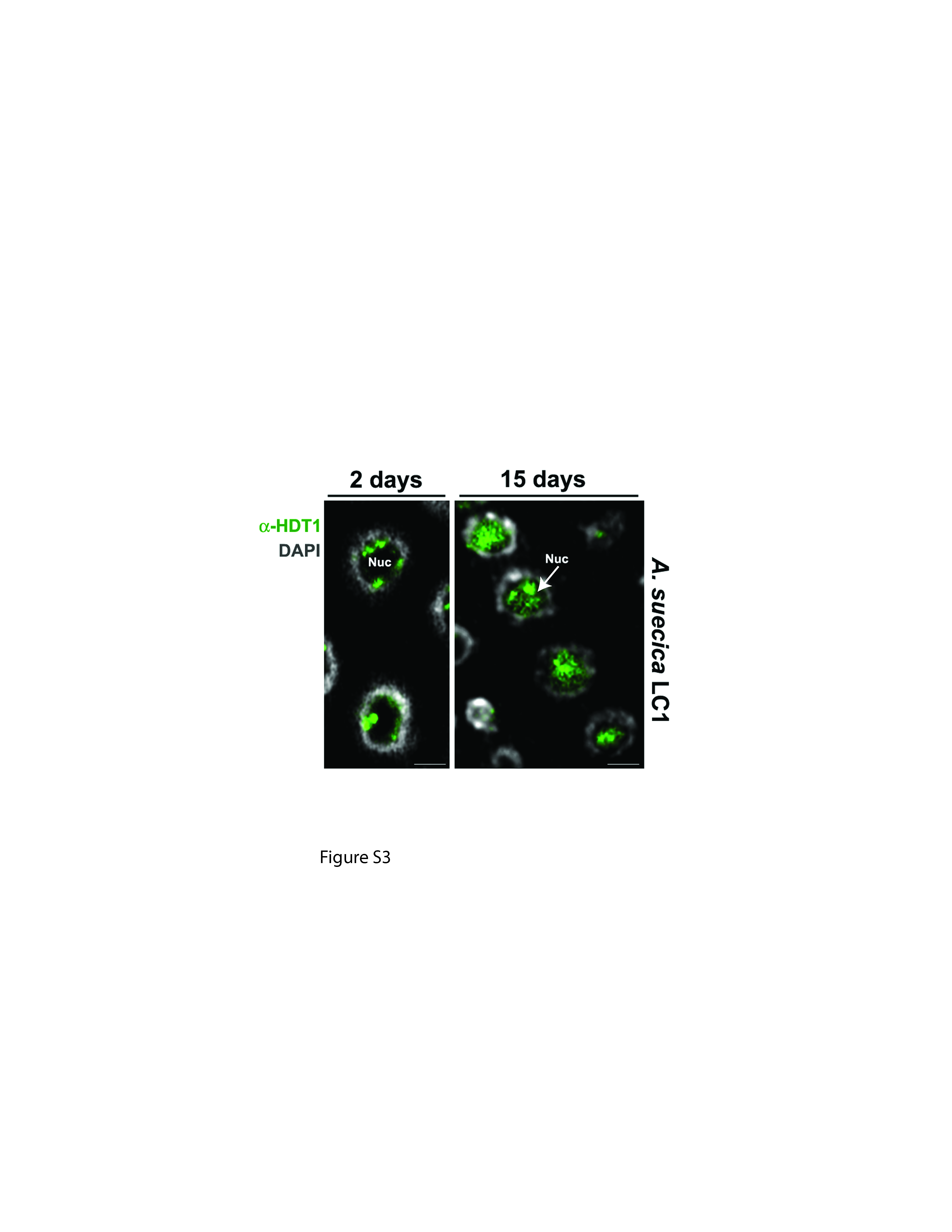

Supplement: Figure S3 — HDT1 immunolocalization patterns in meristematic zone nuclei of whole-mounted 2 and 15 day-old plant root tips. Anti-HDT1 antibody (α-HDT1) signals are in green; DNA counterstained with DAPI is in grey. Each panel shows a single confocal optical section that passes through the centers of the nucleoli (Nuc) in two or more neighboring cells. The nucleoli are the dark regions of the nuclei not stained by DAPI. The size bars correspond to 5 μm. (3.80 MB TIF) [file pone.0001157.s012.tif]

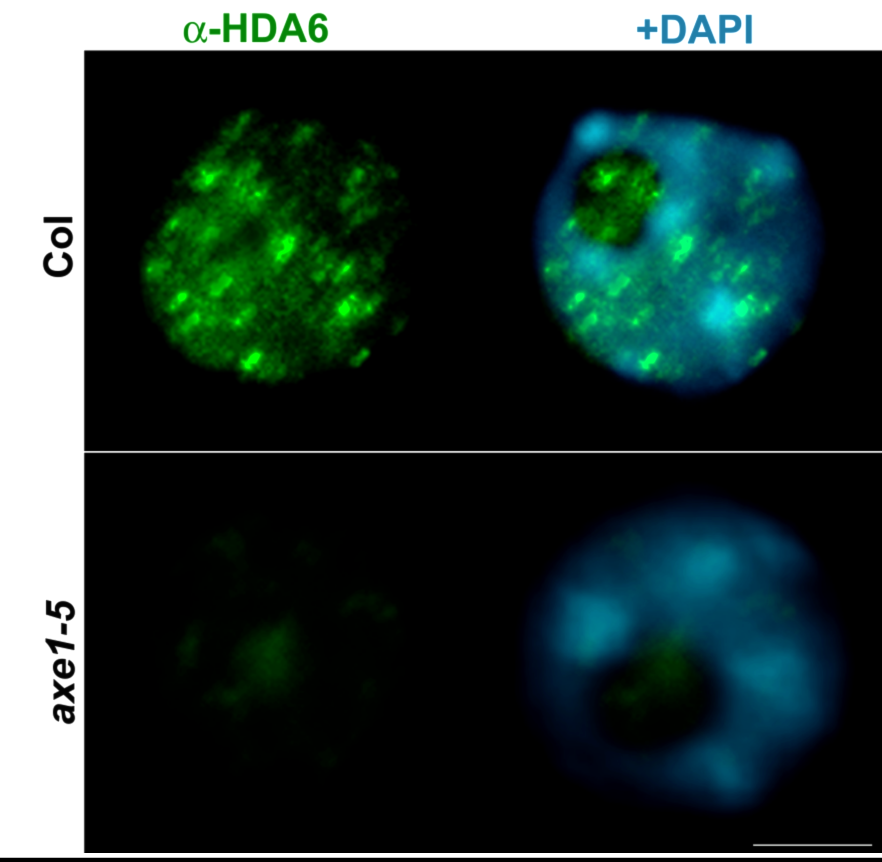

Supplement: Figure S4 — Immunolocalization of HDA6 protein (green signals) in wild-type (Col-0 ecotype) and hda6 (allele axe1-5 allele) mutant A. thaliana. DNA was counterstained with DAPI (blue). The size bar correspond to 5 μm. The severely reduced HDA6 signal in the axe1-5 mutant indicates that the strong signals detected in wild-type nuclei are attributable to HDA6. Moreover, epitope-tagged HDA6 expressed from a transgene and detected by virtue of its epitope tag displays the same localization pattern shown here (see Earley et al., 2006). (0.54 MB TIF) [file pone.0001157.s013.tif]

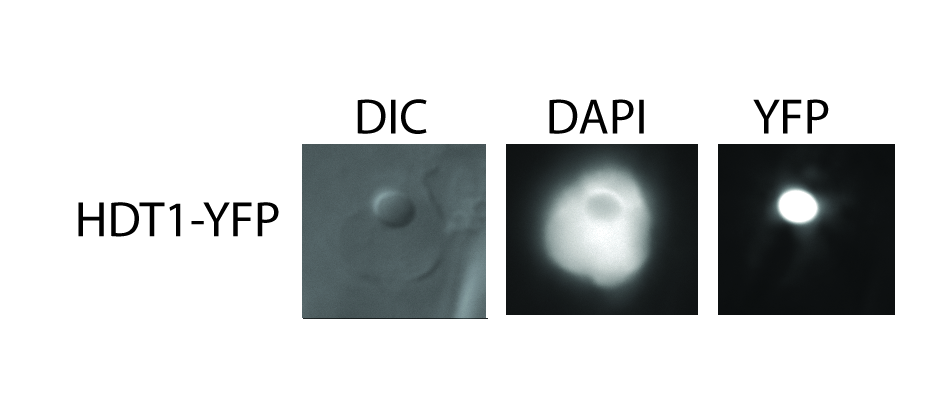

Supplement: Figure S5 — HDT1-YFP localizes to the nucleolus. An intact root of a transgenic A. thaliana plant expressing an HDT1-YFP translational fusion protein was stained with DAPI and subjected to differential interference contrast (DIC) and fluorescence microscopy. The three panels show the nucleus of a single root cell. The nucleolus is readily apparent in the DIC image and corresponds to the region of the nucleus least stained by DAPI. The YFP signal is exclusively localized within the nucleolus, as reported previously (Lawrence et al., 2004). The nucleolar localization of the HDT1-YFP fusion protein supports the nucleolar localization of HDT1 detected using anti-HDT1 antibodies. (2.10 MB TIF) [file pone.0001157.s014.tif]
